# Distinguishing importation from diversification of quinolone-resistant *Neisseria gonorrhoeae *by molecular evolutionary analysis

**DOI:** 10.1186/1471-2148-7-84

**Published:** 2007-06-01

**Authors:** Marcos Pérez-Losada, Keith A Crandall, Margaret C Bash, Michael Dan, Jonathan Zenilman, Raphael P Viscidi

**Affiliations:** 1 Department of Integrative Biology, Brigham Young University, Provo, UT, USA; 2 Department of Microbiology and Molecular Biology, Brigham Young University, Provo, UT, USA; 3 Division of Bacterial, Parasitic and Allergenic Products, Center for Biologics Evaluation and Research, United States Food and Drug Administration, Bethesda, MD, USA; 4 Infectious Diseases Unit, Edith Wolfson Hospital, Tel Aviv, Israel; 5 Division of Infectious Diseases, Department of Medicine, Johns Hopkins University School of Medicine, Baltimore MD, USA; 6 Stanley Division, Department of Pediatrics, Johns Hopkins University School of Medicine, Baltimore MD, USA

## Abstract

**Background:**

Distinguishing the recent introduction of quinolone resistant gonococci into a population from diversification of resistant strains already in the population is important for planning effective infection control strategies. We applied molecular evolutionary analyses to DNA sequences from 9 housekeeping genes and *gyrA*, *parC *and *porB *of 24 quinolone resistant *N. gonorrhoeae *(QRNG) and 24 quinolone sensitive isolates collected in Israel during 2000–2001.

**Results:**

Phylogenetic and eBURST analyses and estimates of divergence time indicated QRNG were introduced on 3 separate occasions and underwent limited diversification by mutation, deletion and horizontal gene transfer. Reconstruction of *N. gonorrhoeae *demography showed a slowly declining effective strain population size from 1976 to 1993, rapid decline between 1994 and 1999, and an increase from 1999 to 2001. This is partially attributable to declining gonorrhea case rates from 1973 to 1994. Additional contributing factors are selective sweeps of antibiotic resistant gonococci and increased transmission from sex workers. The abrupt decline in the mid-1990s heralded an increased incidence of gonorrhea from 1997 to the present. The subsequent increase in effective strain population size since 1999 reflects the increased gonococcal census population and introduction of quinolone resistance strains.

**Conclusion:**

Our study demonstrates the effective use of population genetic approaches to assess recent and historical population dynamics of *N. gonorrhoeae*.

## Background

Resistance to fluoroquinolone antibiotics among *Neiserria gonorrhoeae *strains emerged rapidly in Asia after their introduction in 1989. Recent reports from developed countries have documented clonal spread of quinolone resistant *N. gonorrhoeae *(QRNG) [1-4]. Understanding the origin of an epidemic and dynamics of its subsequent spread are critical in developing effective control strategies. We recently described a high rate of QRNG in Tel Aviv, Israel during 2000–2001 [5]. Genetic analysis of *porB *using molecular probes identified two predominant genotypes among QRNG [6] and pulse field gel electrophoresis demonstrated limited heterogeneity of the resistant strains and clear similarities to resistant strains from southern Israel [7]. These studies suggested recent introduction of QRNG, perhaps from a single source, followed by rapid dissemination through the country. However, not all isolates had the identical genotype raising the question of whether there was diversification of the original strain or whether there were multiple introductions of QRNG strains.

Determining genetic relatedness of isolates can provide insights into the source and pattern of spread of *N. gonorrhoeae *within a community. Previous studies have characterized QRNG by auxotyping/serotyping [3,8], pulse field gel electrophoresis [3,7,9], *porB *gene typing with molecular probes [6], *opa *typing [10,11] or multiantigen sequence typing [4,12]. For our study, we used a MultiLocus Sequence Typing (MLST) method [13,14]. MLST is a technique for characterizing bacterial species using sequences of internal fragments of multiple housekeeping genes [15]. The advantage of MLST for population genetic analysis is that housekeeping genes are presumed neutral evolving genetic markers. Additional insights into evolutionary forces structuring bacterial populations can be obtained from examination of loci subject to selection. Therefore, we also sequenced a partial fragment of *porB *[16], which is under strong positive selection, and segments of *gyrA *and *parC*, which are target loci for fluoroquinolone resistance [17,18]. Based on these analyses we inferred the evolutionary history of isolates from phylogenetic, network and eBURST reconstructions and calculated time of divergence from the most recent common ancestor in order to determine if genetically variant strains diverged before or after the putative time QRNG first appeared in Israel. We also estimated selective pressure on each gene and examined the association of selected sites with the ciprofloxacin resistance phenotype. Finally, using a novel analytical approach from population genetics, we estimated the past population dynamics of *N. gonorrhoeae *in Israel.

## Results

An increase in the incidence of gonorrhea was observed in Israel in 1998, accompanied by the appearance of quinolone resistant *N. gonorrhoeae* (QRNG) isolates [5]. The incidence of gonorrhea peaked in  2002 and declined sharply thereafter. The rate of isolation of QRNG  strains also declined after 2001. During the epidemic period, QRNG  strains were detected in several parts of Israel.  Pulse field gel  electrophoresis analysis of isolates from the Negev region in southern  Israel, Jerusalem, Haifa, and Tel Aviv showed that all the QRNG strains were closely related [7].  In the Tel Aviv area, there were 200 cases of gonorrhea in 2000 and 325 cases in 2001.  The isolates for the present  study were obtained from January 2000 through October 2001 in Tel Aviv,  Israel [5]. Of 80 isolates collected during this time period that were  previously genotyped by molecular probes for the variable regions of the *porB* gene [6], we selected 24 fluoroquinolone resistant and 24 sensitive strains for more detailed genotyping by sequencing of fragments of multiple housekeeping genes.

**Figure 1:**
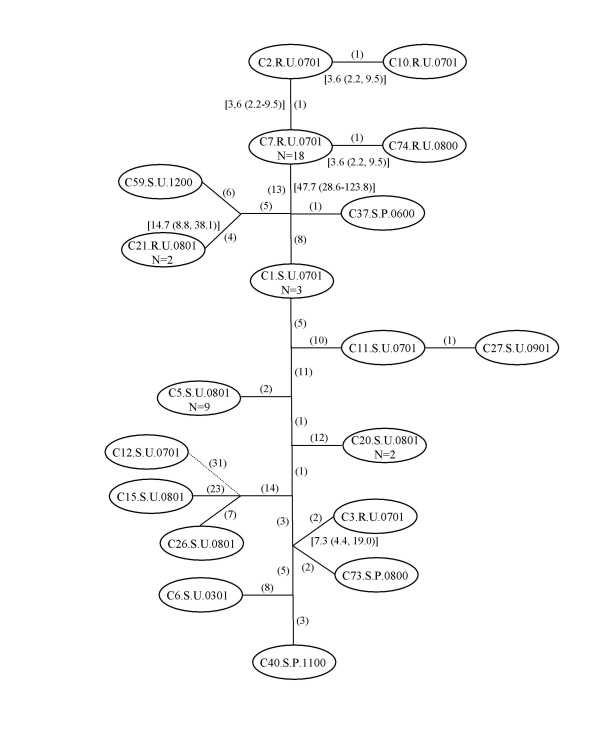
Statistical parsimony network of the 9 housekeeping genes included in the MultiLocus Sequence Typing (MLST) scheme. Number of steps is indicated between parentheses. Mean divergence time estimate in years and 95% highest posterior density (HPD) limits are shown in brackets. N = number of sequences. Dotted line indicates a connection <95% parsimony probability (30 steps). C1.S.U.0701 = C9.S.U.0701 and C23.S.U.0801; C5.S.U.0301 = C38.S.P.0400, C41.S.P.1200, C51.S.F.0101, C55.S.U.1200, C57.S.U.1200, C64.S.U.1200, C68.S.U.0900 and C78.S.U.0700; C20.S.U.0801 = C22.S.U.0801; C7.R.U.0701 = C14.R.U.0701, C28.R.P.1001, C29.R.P.0901, C32.R.U.0800, C33.R.F.0800, C35.R.P.0600, C36.R.P.0700, C39.R.P.0300, C45.R.F.0900, C46.R.P.0700, C54.R.U.1200, C58.R.U.1200, C61.R.U.1100, C63.R.U.1000, C66.R.U.1200, C70.R.U.0800 and C79.R.P.0700; C21.R.U.0801 = C24.R.U.1001.

Phylogenetic relationships among the ciprofloxacin sensitive and resistant strains based on 9 housekeeping genes were estimated using the statistical parsimony procedure and graphically depicted as a network of gene genealogies (Figure 1). The lines on the network indicate mutational connections among the unique genotypes with the number of substitutions separating these sequences in parentheses adjacent to the line. Genotypes were designated by strain number (CX), ciprofloxacin susceptibility (R = resistance, S = sensitive), contact information (P = paid commercial sex worker, F = non-paid sex worker, U = unknown), and month and year of isolation. There was no clustering of strains by contact information or date of isolation. Eighteen of 24 resistant strains had the identical sequence or genotype, represented in the genealogy by C7.R.U.0701. C2.R.U.0701 and C74.R.U.0800 differed by one mutational step from the most common genotype, and C10.R.U.0701 differed by one nucleotide substitution from C2.R.U.0701. Thirteen mutational steps separated these resistant strains from the most recent common ancestor for all the other strains in the data set. C21.R.U.0801 and C24.R.U.1001 had identical genotypes and were separated by 10 mutational steps from the next most closely related strains, which were sensitive isolates. The remaining resistant isolate, C3.R.U.0701, was distantly related to both the other resistant strains and was separated by 4 mutational steps from the most closely related sensitive strains. As a group, the sensitive strains were more genetically diverse than the resistant strains with 13 unique genotypes among 24 strains. However, 9 strains had the same genotype, which is represented in the network by C5.S.U.0801. For the resistant strains, we calculated time of divergence from the most recent common ancestor, and mean years and 95% highest posterior density (HPD) limits are shown in parentheses adjacent to the line connecting genotypes. C2R.U.0701 and C74.R.U.0800 diverged from the majority resistant genotype on average in early 1998 or 1997, respectively, and possibly as recently as mid-1999 or mid-1998, respectively. C10.R.U.0701 is also likely to have diverged from the other resistant strains sometime in 1998 or 1999.

**Figure 2:**
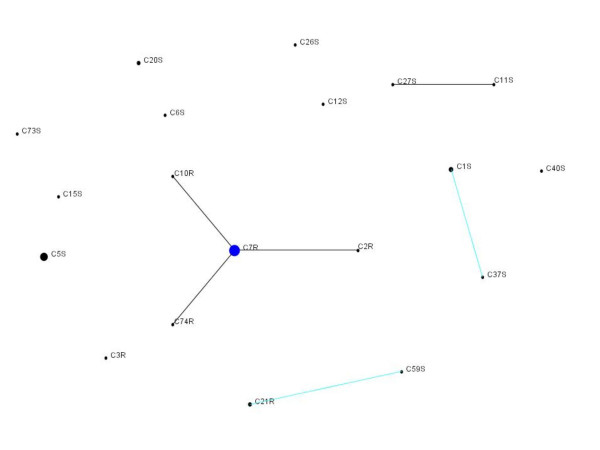
eBURST diagram displaying the relatedness of 48 Israeli isolates. All the STs are displayed in a single diagram using settings for a population snapshot. ST names correspond to the strain number (CXX), followed by the letter R (resistant) or S (sensitive) to indicate quinolone sensitivity. The area of each circle corresponds to the number of isolates. Black lines connect single locus variants and turquoise lines connect double locus variants. A blue circle indicates the predicted founder of a complex with three or more STs.

The allelic profiles of the 48 strains produced 13 different STs. The eBURST program assigned these STs to four clonal complexes (Figure 2). The majority resistance genotype (represented by C7.R.) was grouped with C2.R, C74.R and C10.R, supporting the recent evolutionary relationships between these strains revealed by the statistical parsimony procedure. C11.S and C27.S were single locus variants of each other, consistent with the close evolutionary relationship (one mutational step) found by the statistical parsimony procedure. Two pairs of double locus variants were identified; C21.R (representative of 2 resistant strains) and C59.S, and C37.S and C1.S (representative of 3 sensitive strains). In the statistical parsimony analysis these strains were separated by 10 and 9 mutational steps, respectively. Although C3.R and C73.S were separated by 4 mutational steps they did not form a clonal complex by eBURST analysis. All the remaining strains were separated by more than 10 mutational steps in the parsimony analysis.

**Figure 3:**
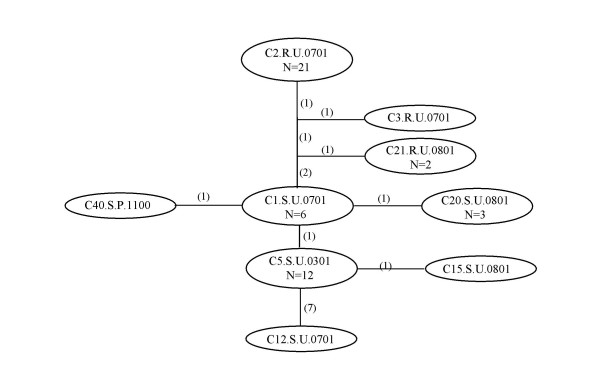
Statistical parsimony network of two quinolone resistance genes, *gyrA *and *parC *Number of steps is indicated between parentheses. N = number of sequences. C1.S.U.0701 = C9.S.U.0701, C11.S.U.0701, C23.S.U.0801, C27.S.U.0901, and C37.S.P.0600. C5.S.U.0301 = C6.S.U.0301, C26.S.U.0801, C38.S.P.0400, C41.S.P.1200, C51.S.F.0101, C55.S.U.1200, C57.S.U.1200, C64.S.U.1200, C68.S.U.0900, C73.S.P.0800, and C78.S.U.0700. C20.S.U.0801 = C22.S.U.0801 and C59.S.U.1200. C21.R.U.0801 = C24.R.U.1001. C2.R.U.0701 = C7.R.U.0701, C10.R.U.0701, C14.R.U.0701, C28.R.P.1001, C29.R.P.0901, C32.R.U.0800, C33.R.F.0800, C35.R.P.0600, C36.R.P.0700, C39.R.P.0300, C45.R.F.0900, C46.R.P.0700, C54.R.U.1200, C58.R.U.1200, C61.R.U.1100, C63.R.U.1000, C66.R.U.1200, C70.R.U.0800, C74.R.U.0800, and C79.R.P.0700.

The network of genotypes of the fluoroquinolone resistance genes (Figure 3) showed a much lower level of genetic diversity over all than that of the housekeeping genes. Twenty-one of 24 resistant strains had the identical genotype, with only those three strains that were also distantly related at the housekeeping gene loci exhibiting different quinolone resistance genotypes. Among the quinolone sensitive strains, there were six genotypes, with 50% of strains having the identical genotype, which is represented in the network by C.5.S.U.0301.

**Figure 4:**
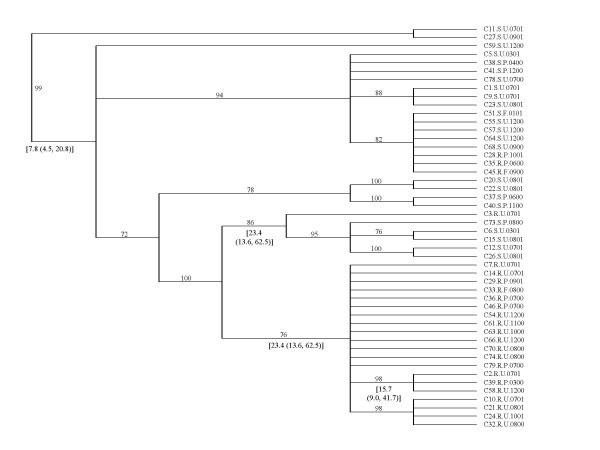
Bayesian 50% majority-rule consensus tree of *porB *sequences. Mean divergence time in years and 95% highest posterior density (HPD) limits are indicated in brackets. Clade support posterior probabilities (if ≥ 50%) are shown over the branches.

The phylogeny for *porB *sequences was estimated by the Bayesian method and evolutionary relationships among genotypes are displayed as a 50% majority-rule consensus tree (Figure 4). There was no clustering of strains by contact information or date of isolation. Thirteen resistant strains had the identical *porB *sequence. Six strains differed from the majority clade by a 3–9 base pair deletion. C2.R.U.0701 differed by a 3 bp deletion and two nucleotide substitutions. The majority clade was estimated to have diverged from the most recent common ancestor approximately 23 years ago. C2.R.U.0701 formed a branch sister to the majority clade with a mean divergence time of 16 years. C3.R.U.0701 was distantly related to the other *porB *sequences in the data set. Three resistant strains, C28.R.P.1001, C35.R.P.0600 and C45.R.F.0900, all from men who reported contact with sex workers, had the same *porB *sequence as that of five sensitive strains. The five sensitive strains shared a common housekeeping and fluoroquinolone resistance genotype, which was also the most prevalent genotype among the sensitive strains (represented by C5.S.U.0801 on the housekeeping and quinolone resistance gene genealogies). The three resistant strains were identical at all 9 housekeeping and 2 fluoroquinolone resistance gene loci to the most common resistant genotype and shared no alleles at these loci in common with the five sensitive strains.

To determine whether positive selection has been a force in the evolution of *N. gonorrhoeae*, positively selected sites were identified by estimating the per site nonsynonymous/synonymous rate ratio and by evaluating changes in amino acid properties (Table 1). With the exception of *glnA *and *serC*, there were very few positively selected sites (1–3) in the housekeeping genes and generally the same amino acid was found with high frequency among the resistant and sensitive strains. At five sites in *glnA *the amino acid found most often in the resistant strains was a low frequency amino acid in the sensitive strains. At amino acid position 284 in *pilA *all the sensitive strains had a threonine and 23 of 24 resistant strains had an alanine. Two positively selected sites were identified in *gyrA *(amino acid position 91 and 95) and one site in *parC *(position 86). The resistant strains had mutations, Ser91Phe and Asp95Asn in *gyrA *and Asp86Asn in *parC*, that are consistent with quinolone resistance determining mutations identified in other studies. Seventeen positively selected sites were identified in *porB *and many sites were polymorphic. A previous study showed that a single amino acid mutation to Lys at residue 120 of the Por IB protein confers full intermediate level resistance to penicillin and tetracycline and a single Asp mutation at either position 120 or 121 (22 and 23 in our shorter sequences) confers partial resistance [19]. By our analysis, both amino acid residues are under strong positive selection. A single Lys was found at position 120 in all 24 resistant strains and 15 sensitive strains, and 2 sensitive strains had an Asp at either position 120 or 121 (Table 2).

**Table 1:** Positively selected amino acid sites (AA site). Sites based on d_N_/d_S _> 1 under PAML and change in amino acid property by TREESAAP (TS) and absolute amino acid frequencies at those sites in susceptible (AA-S), resistant (AA-R), C3 strain (AA-C3R), and C21\C24 strains (AA-21R/24R). All AA sites including gaps were not included

Locus	AA site^a ^(PAML)	AA site (TS)	AA-S	AA-R	AA-C3R	AA-21R/24R
*abcZ*	152	152	14K-10E	21K	E	E
*fumC*	310		6M-18I	3I-18M	M	I
*gdh*	253		5G-19S	21S	S	S
*glnA*	12	12	3K-21E	21K	E	K
*glnA*	14	14	3K-21E	21K	E	K
*glnA*	42	42	3G-21D	21G	D	G
*glnA*	51	51	3Q-21P	21Q	P	Q
*glnA*	68		3Q-21E	21Q	E	Q
*glnA*	91		1L-23I	21I	I	I
*glnA*		151	1I-23T	21T	T	T
*gnd*	301		2R-22K	21K	K	K
*gnd*	350	350	10D-14G	1D-20G	D	G
*gyrA*	91	91	23S-1T	21F	F	F
*gyrA*	95	95	23D-1N	21N	N	G
*parC*	86	86	24D	21N	N	N
*pilA*	181	181	6G-18D	21D	G	D
*pilA*	273	273	1E-23G	21G	G	E
*pilA*	284		24T	21A	T	A
*ppk*	551		4Q-20R	21R	R	R
*pyrD*		66	1K-23E	21E	E	E
*pyrD*		72	1D-23G	21G	G	G
*serC*	37	37	9P-15S	21S	S	P
*serC*	40	40	9A-15E	21E	E	A
*serC*	164		3Q-21R	21R	R	R
*serC*	165	165	3I-21E	21E	E	E
*serC*	168		3K-21R	21R	R	R
*serC*		193	1G-23D	21D	D	D
*porB*	22	22	15K-7G-2D	23K	1K	-
*porB*	23	23	14D-7A-1G-2S	23D	1D	-
*porB*		35	22G-2D	23G	1G	-
*porB*		36	8N-12D-4E	20N-3D	1N	-
*porB*		42	4K-20G	23G	1G	-
*porB*	45		5Q-2E-2R-15K	23K	1Q	-
*porB*	89	89	2Q-22R	23R	1Q	-
*porB*	91		4N-5S-15G	3G-20N	1S	-
*porB*		111	2M-22I	23I	1I	-
*porB*	117	117	2L-3V-2Y-2Q-1D-2S-1T-1D-10F	3F-20V	1A	-
*porB*	120	120	13V-7I-2S-1T-1M	3V-20I	1I	-
*porB*	157	157	2Q-22G	23G	1G	-
*porB*	160	160	5A-2L-17T	3T-20A	1A	-
*porB*	161	161	3R-2V-2T-17W	3W-20R	1R	-
*porB*	162	162	2G-1S-21R	23R	1R	-
*porB*	163	163	5D-2V-17A	23A	1A	-
*porB*	176		4V-20A	23A	1A	-

^a ^AA site position numbers based on translated amino acid sequence of gene from strain FA1090, with the exception of *porB*.

**Table 2:** Amino acid mutations at residues 120 and/or 121 that confer resistance to penicillin and tetracycline in *N. gonorrhoeae*.

porB	Lys (120)	Asp-Asp (120–121)	Asp (120 or 121)	Other AA (120 or 121)	Total
R strains	24	0	0	0	24 (100%)
S strains	15	0	2	7	17 (70.8%)

**Figure 5:**
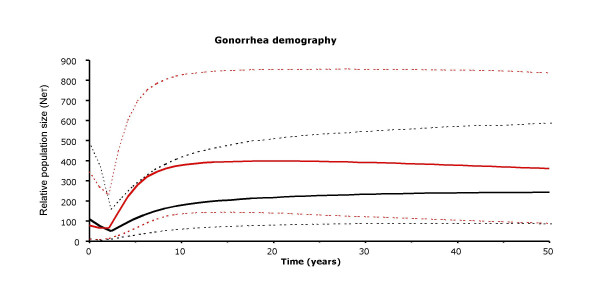
*N. gonorrhoeae *population dynamics in Israel over the past quarter century. Relative genetic population size (Neτ, where Ne is effective population size and τ is number of generations) was estimated from 9 housekeeping genes (red lines) and the *porB *gene (black lines) with solid line showing the mean estimate and the dashed lines the 95% highest posterior density (HPD) limits.

The historical demography of *N. gonorrhoeae *in Israel over the past 24 years was estimated from the housekeeping gene and *porB *sequences using a Bayesian MCMC method that allows the inference of past population dynamics from contemporary sequences (Figure 5). After decreasing slowly from 1976 to approximately 1993, the effective strain population size declined sharply from 1993 to 1999. However, since 1999, it has been flat to slightly increasing. The census population of gonococcal infections in Israel, represented by the clinical case-incidence rate per 100,000 persons, declined from 40 in 1970 to 4 in 1987 and to 0.9 in the mid 1990's [20]. In 1999, the rate rose to 3.9 and further increased to 8.3 in 2000. The genetic data support this demographic information on the declining infection rate since the 1970s and the recent rise of infection rates since the late1990's

## Discussion

Quinolone-resistant *Neisseria gonorrhoeae *(QRNG) are currently a worldwide problem. In many developed countries QRNG infections reflect a combination of importation and endemic spread. Importation can be inferred from a medical history of having had sex partners abroad or contact with someone who did; however, patient reports may not be reliable, particularly if there has been sex with anonymous partners or commercial sex workers. Genotyping has been applied to QRNG and the detection of clusters of identical strains is thought to reflect endemic transmission, while detection of multiple, heterogeneous isolates is characteristic of imported strains. However, because gonococci undergo rapid genetic diversification [21-23], a clonal population may not persist for long periods of time. Thus, sampling from the general population of patients with QRNG may yield a genetically complex sample reflecting the diverse epidemiology of QRNG infections and the evolution of *N. gonorrhoeae*.

In order to distinguish between importation and diversification, we applied molecular evolutionary analyses to a recent outbreak of QRNG in Israel. Advantages of Israel for such studies are that the country is small, contained, and has a good health care system, and gonorrhea is a notifiable disease. Our phylogenetic analyses suggested that QRNG were imported into Israel on at least three separate occasions, resulting in an isolated infection by strain C3R, two infections by the genetically identical strains, C21R and C24R, and 21 infections with the remaining resistant strains. In support of importation as the source of these infections, the housekeeping gene genealogy showed that C3R and C21/24R were distantly related to the other resistant strains and all the resistant strains had diverged from the most recent common ancestor prior to the first known appearance of QRNG in Israel. These strains also differed at the quinolone resistance loci but an accurate divergence time could not be calculated due to their low genetic variation. C3R was also distantly related to the other resistant strains at the *porB *locus. Since quinolones were introduced into clinical practice in Israel in the mid 1990s, we cannot entirely exclude the possibility of antibiotic selection pressure acting on indigenous Israeli strains. Antibiotic selection pressure is unlikely to explain the emergence of the majority resistant clone since it was distantly related to all the sensitive Israeli strains we sampled. However, C3R may have diverged as recently as 4.4 years earlier from C73S, placing the split close to the mid 1990s. Our estimate of the lower limit for divergence time between C21R/C24R and C59S is 8.8 years. In the eBURST analysis, C21R/C24R and C59S were double locus variants, an observation that suggests they may be evolutionarily closely related. Additional sampling of sensitive strains from the 1990s may help resolve the uncertainty regarding the origins of the C3R and C21R/C24R strains.

We also found evidence to support genetic diversification within the population of resistant strains after they entered Israel. Strains C2R, C10R and C74R were within one mutational step of the clonal population of resistant strains at the housekeeping gene loci and could have diverged from a common ancestor within the time period that resistant strains have circulated in Israel. The lower limit for the divergence time would place these events in 1998 or 1999. The eBURST analysis grouped these strains as a clonal complex. By definition clonal complexes are presumed to share a recent common ancestor. Six strains differed from the majority of resistant strains at the *porB *locus by a 3–9 bp deletion. Although a divergence time cannot be estimated for deletions, because the strains did not accumulate any substitutions, the deletions must be relatively recent events. C2R differed at the *porB *locus from the majority of resistant strains by a deletion and two nucleotide substitutions. Because the estimate of divergence time suggested a split prior to the early 1990s, we cannot entirely exclude the possibility that this strain represents a separate importation. Three strains, C28R, C35R and C45R, did not differ from the other resistant strains at the 9 housekeeping and 2 quinolone resistance gene loci but they had a variant *porB *sequence that was prevalent among sensitive strains in Israel. The acquisition of a new *porB *sequence by these strains is likely the result of horizontal gene transfer and may be a recent event. Of note these subjects reported having sex with commercial sex workers who are likely to harbor multiple gonococcal strains, which would increase the chances for horizontal gene transfer.

Positive selection can have a strong impact on bacterial evolution; therefore, we analyzed the nucleotide sequences of the resistant and sensitive strains for positively selected sites. As expected, positively selected sites were identified in the quinolone resistance genes, *gyrA *and *parC*, and the resistant strains had mutations that have previously been shown to be determinants of quinolone resistance. Although the three resistance genotypes did not share a common ancestor they had common Ser91Phe *gyrA *and Asp86Asn *parC *mutations, demonstrating convergent evolution of these drug resistant mutations. The housekeeping genes, except for *glnA *and *serC*, had a paucity of positively selected sites consistent with the expectation that these genes are not under strong positive selection. No mutations at positively selected sites in the 9 housekeeping genes were significantly associated with QRNG. In contrast, 17 positively selected sites were identified in the *porB *sequence and many sites were highly polymorphic. This is not surprising given the selection pressure on this gene from the immune system and antibiotic treatment. Mutations to charged amino acids at two positively selected sites, corresponding to residues at position 120 and 121 of the *N. gonorrhoeae *PIB porin, have been associated with resistance to penicillin and tetracycline [19] and these mutations were found in 100% of the QRNG strains, although they were also present in a high proportion of the quinolone sensitive strains.

Changes in effective population size over time can provide insights into the epidemiology of gonorrhea. Effective population size is a complex evolutionary parameter reflecting changes in the actual population size, excluding genetically equivalent organisms, and also the influence of environmental and host factors on the ability of individual strains to contribute to future generations. Our analysis of population dynamics of *N. gonorrhoeae *over the past quarter century in Israel revealed a slow decrease in the effective strain population size from 1976 to 1993, a steeper decline from 1993 to 1999 and then a rise from 1999 to the present. Since the steepest decline in the census population of gonococcal isolates occurred between 1981 and 1987 [20], it is likely to have played only a small part in the decrease in the effective population size during the 1990s. A potentially important factor in the decline is the appearance in Israel, as elsewhere in the world, of penicillinase-producing *N. gonorrhoeae *(PPNG) and chromosomally mediated penicillin resistance during the late 1980s and early 1990's. A selective sweep by antibiotic resistant strains would be expected to decrease genetic complexity of the gonococcal population and thus cause a decrease in effective strain population size. Behavioral changes, such as increased transmission from sex workers (see below), could also reduce genetic diversity as a result of common source outbreaks with genetically related strains. The increase in the census population of gonococcal isolates in Israel since 1998 has been attributed primarily to an increase in the sex worker population, especially of foreign workers from countries with high rates of gonorrhea [20]. If this interpretation is correct, then the decrease in effective strain population size in the mid 1990s may have been a harbinger of the subsequent increase in rates of gonorrhea in Israel. The rise in effective strain population size since 1999 is consistent with the increase in the census population and also with the recent introduction and spread of QRNG. The imported QRNG strains differed from prevalent quinolone sensitive strains at many loci. Additionally, these strains appear to have evolved rapidly through point mutation, recombination and horizontal gene transfer. All these factors would be expected to increase the genetic diversity of the pool of *N. gonorrhoeae *strains in Israel and thus increase the effective strain population size. A limitation of our reconstruction of population dynamics is the small sample size (48 strains). However, we compensated for the small number by sampling multiple loci (11 gene fragments). A limitation of the BEAST method is the assumption of no recombination. We did not detect recombination in our data set, as indicated by RDP2 [24], and, moreover,, the correlation between the population genetic inference and the prevalence of *N. gonorrhoeae *during the most recent time period demonstrates the utility of the analytical approach, as suggested before [25].

## Conclusion

We demonstrate the effective use of population genetic approaches to assess recent and historical population dynamics of *N. gonorrhoeae*. Our analysis indicates that QRNG are likely to have entered Israel on at least three occasions and resistant strains have evolved at multiple loci during the 3–4 years that these strains have circulated in the country. The emergence of these strains in Israel has lead to an increase in genetic diversity at antigenically important loci and an increase in the effective strain population size, as well as an increase in infection rates. Reconstruction of evolutionary relationships and estimation of divergence time in conjunction with traditional gonococcal surveillance data can assist in understanding the development, impact and public health importance of QRNG.

## Methods

### Description of isolate collection and sequence determination 

The isolates for the present study were obtained from January 2000  through October 2001 in Tel Aviv, Israel [5,26].  The majority of  isolates were a random sample of male urethral isolates.  Of 80 isolates  collected during this time period that were previously genotyped by  molecular probes for the variable regions of the porB gene [6], we  selected 24 fluoroquinolone resistant and 24 sensitive strains for more  detailed genotyping by sequencing of fragments of multiple housekeeping  genes.  For isolates with an identical porB genotype, only a  representative strain was typed.  All patient identifiers associated  with isolates were removed prior to typing.

Genomic DNA was isolated as described previously [6]. Partial regions of the following 9 core housekeeping genes were sequenced: *abcZ*, *fumC*, *gdh*, *glnA*, *gnd*, *pilA*, *ppk*, *pyrD*, and *serC *[14]. Fragments of *gyrA *and *parC*, encompassing the fluoroquinolone resistance-determining regions, and a segment of *porB*, spanning hypervariable loops 3 to 6, were also sequenced. PCR reactions and nucleotide sequencing of PCR products were performed as described previously [25].

### Genetic analysis

A total of 576 DNA sequences were used for the genetic analyses; 432 housekeeping gene sequences, 48 *porB *sequences and 48 *gyrA *and *parC *gene sequences. Unique sequences were deposited in GenBank under accession numbers [GenBank: EF014714–EF014722 and EF016649–EF016670]. Sequences were translated into amino acids using the universal reading frame in MacClade 4.05 [27] and then aligned in ClustalX [28] using the default options. The 9 housekeeping genes and the 2 fluoroquinolone resistance genes were separately concatenated into two sequences for each isolate.

Evolutionary relationships among *porB *gene sequences were assessed using the Bayesian approach in MrBayes v3.0 [29]. Models of nucleotide substitution were assessed using a maximum likelihood approach [30] with best-fit models selected by Modeltest v3.06 [31] using Akaike information criterion [32]. The substitution model GTR+Γ+I was selected as the best-fit model of molecular evolution. The *porB *sequences exhibited length polymorphism. We introduced that information into the Bayesian analysis coupled with the *porB *sequence variation by coding each gap string as a different character. Evolutionary relationships among resistant and susceptible housekeeping and fluoroquinolone resistance gene sequences were assessed using the method of statistical parsimony [33] as implemented in the software package TCS v1.13 [34]. Statistical parsimony has been demonstrated to significantly outperform traditional phylogenetic approaches when the level of divergence among sequences is low [35], as it is for housekeeping genes. Moreover, this method allows for moderate levels of recombination when estimating reticulate connections among genotypes.

Recent evolutionary relationships among the housekeeping gene sequences were analyzed using the eBURST algorithm [36]. First, allele numbers were assigned to each of the nine housekeeping loci and the allele numbers for all nine genes of each isolate were assembled into a string of integers giving the allele profile for each isolate. Each unique allelic profile was assigned a sequence type (ST) number. The STs and their allelic profiles are the input data used by eBURST. The program groups STs into clonal complexes. A criterion of seven shared alleles out of the 9 alleles examined was used to define a clonal complex. Thus, within a clonal complex, the STs must share seven or more alleles with at least one other ST of the clonal complex. Two STs that differ at one locus are referred to as single locus variants and those that differ at two loci as double locus variants.

The past population dynamics of *N. gonorrhoeae *in Israel was inferred using the Bayesian skyline plot model [37] as implemented in BEAST v1.3 [38] under the GTR+Γ+I model for both the 9 housekeeping and *porB *genes. Because BEAST assumes no recombination, before performing the analysis, we tested for the presence of recombinant sequences and gene regions in all the data sets by using RDP2 [24]. Uniform prior distributions were used for the mean substitution rates. Lower and upper limits and mean of these distributions were obtained from an extensive *N. gonorrhoeae *data set from Baltimore recently analyzed by our group [25]. These substitution rates were used for estimating divergence times of the resistant Israeli strains (see below). The Bayesian Markov chain Monte Carlo (MCMC) output generated by BEAST was analyzed in Tracer v1.3 [39].

Mean divergence times (and 95% posterior probability intervals) of the most common recent ancestor of the resistant strains were estimated on the 9 housekeeping gene-TCS network and the *porB*-Bayesian tree assuming a substitution rate distribution previously estimated under a relaxed clock model [25]. We used the following mean substitution rates (and 95% posterior probability intervals) measured as the number of substitutions per site with the per gene and year rates given in parentheses: 9 housekeeping genes = 0.2723 (0.105, 0.4538) and *porB *= 0.128 (0.048, 0.221).

We inferred the extent of natural selection by estimating the ratio of nonsynonymous to synonymous substitutions, ω (= d_N_/d_S_) per site using the codon-based nested models M7 (beta) and M8 (beta and ω) of Yang et al. [40] as implemented in the PAML package [41]. Model likelihood scores were compared using a likelihood ratio test. When ω is greater than 1 in M8 positively selected sites can be inferred. We applied the empirical Bayesian approach [42] to identify the potential sites under diversifying selection as indicated by a posterior probability (pP) > 0.95. Additionally, we used the approach of McClellan et al. [43] for inferring sites under positive destabilizing selection (i.e., selection that results in radical structural or functional shifts in local regions of the protein; comparable to adaptive selection in PAML) in terms of 31 quantitative amino acid properties. We followed the procedure in McClellan [44] in combination with a recently described analytical algorithm [13] implemented in TreeSAAP ver3.2 [45].

## Authors' contributions

MP-L participated in the design of the study, carried out the molecular evolutionary analyses and drafted the manuscript. KAC participated in the conception and design of the study, assisted with molecular evolutionary analyses and helped to draft the manuscript. MCB helped to draft the manuscript. MD supervised collection of gonococcal isolates and helped to draft the manuscript. JZ helped to draft the manuscript. RPV participated in the conception and design of the study, supervised generation of nucleotide sequence data, assisted with molecular evolutionary analyses and drafted the manuscript. All authors read and approved the final manuscript.
